# Editorial: Molecular Function and Regulation of Non-coding RNAs in Multifactorial Diseases

**DOI:** 10.3389/fgene.2016.00009

**Published:** 2016-02-19

**Authors:** Mohammadreza Hajjari, Seyed Javad Mowla, Mohammad Ali Faghihi

**Affiliations:** ^1^Department of Genetics, Shahid Chamran University of AhvazAhvaz, Iran; ^2^Department of Molecular Genetics, Faculty of Biological Sciences, Tarbiat Modares UniversityTehran, Iran; ^3^Department of Psychiatry and Behavioral Sciences, Center for Therapeutic Innovation, University of Miami Miller School of MedicineMiami, FL, USA

**Keywords:** long non-coding RNA (lncRNA), microRNA (miRNA), multifactorial disease, biomarker discovery, non-coding RNA (ncRNA)

Most of the parts in the human genomic DNA do not produce any proteins. They encode functional RNA molecules, which are not translated, and are thus named as non-coding RNAs (ncRNAs; Cammaerts et al.; Hajjari et al., 2014). Depending on the length and the function of these RNAs, they are categorized into different types such as microRNAs (miRNAs) and long non-coding RNAs (lncRNAs). Different studies have shown that these RNAs have crucial roles in cellular and molecular mechanisms. Considering their function, it is believed that the dysregulation of ncRNAs is involved in different diseases, especially such complex ones as cancers, neurological disorders, and cardiovascular diseases (Nouraee and Mowla; Merelo et al.; Hajjari et al., 2014). Accordingly, some researchers believe that these molecules can help with the diagnosis and the treatment of complex diseases. However, we have still a limited understanding of the functions of these molecules, and the different aspects of ncRNAs are still to be discovered in future. The above consideration was the motivation for the production of the topic entitled “Molecular function and regulation of non-coding RNAs in multi-factorial diseases.” We believe that the articles in this issue and the attributed e-book can provide the interesting viewpoints for the researchers interested in this topic.

miRNAs are small RNAs with about 20–24 nucleotides length and function in post transcriptional regulation of gene expression. They usually destabilize and repress target RNAs via binding to 3′UTRs, 5′UTRs or the coding sequences of the transcripts (Lytle et al., 2007; Qin et al., 2010). miRNAs have been demonstrated to play major roles in a wide range of developmental processes as well as diseases. To understand the role of miRNA pathogenesis in different diseases such as cancers, two different strategies including the expression analysis and the genetic approaches are proposed (Reviewed in Cammaerts et al.). As such, the miRNAs are currently considered as new potential biomarkers for different diseases (Angelini and Emanueli). Furthermore, some reports have interestingly shown the plasma and saliva miRNAs as potentially sensitive and specific biomarkers (Lin et al.; Khoo et al., 2012). Nonetheless, there are still some concerns with the functionality of miRNAs that need to be addressed in future.

By modulating different targets or pathways, miRNAs have been quickly considered as potential therapeutic molecules for some diseases such as cancer (reviewed in Naidu et al., 2015) and cardiovascular ones (Nouraee and Mowla). miRNA-based therapeutics mainly focuses on modulation of the miRNA expression levels (Misso et al., 2014). MRX34, an agent mimicking miR-34 and developed by Mirna Therapeutics, has progressed into phase I clinical trials (identifier: NCT01829971, Currently recruiting participants, Estimated study completion date: Dec 2016). The results may hopefully provide more insights for the researchers. In spite of different researches, there are still some challenges in designing a suitable carrier for targeting miRNAs into the desired cells. Bakhshinejad reports the studies proposing the nanocarriers as the potential tools to overcome delivery problems concerned with miRNA-based pharmaceutical tools (Bakhshinejad).

lncRNAs, the RNAs longer that 200 nucleotides, comprise the largest proportion of the human transcriptome. The biology of lncRNAs seems more complicated in comparison to miRNAs. These RNAs act through more different pathways and modes of action within the cell. They function at transcriptional and post-transcriptional levels, in the cytosol and nucleus through cis and trans-regulatory mechanisms (Hajjari et al., 2014; Angrand et al.). Different lncRNAs such as *HOTAIR, H19, MEG3, ANRIL, HULC*, and *XIST* are transcribed within the genome and play important roles in different cellular pathways (reviewed in Hajjari et al., 2014). Multiple lines of observation increasingly associate the mutations and the dysregulations of lncRNAs to some human diseases such as cancer (reviewed in Angrand et al.; Hajjari et al., 2014). On the current subject matter, Shahryari et al. have provided some evidence showing that some lncRNAs such as *SOX2OT* are also involved in pluoripotency (Shahryari et al.). Peschanscky et al. have also described the novel facets of *FMR4* lncRNA functionality and its relation to neurodevelopment (Peschansky et al.).

The growing evidence of the dysregulated lncRNAs not only introduces a new layer of complexity in the molecular mechanism of human diseases, but also opens up the opportunity to use lncRNAs as therapeutic targets and biomarkers. The prominent example of lncRNAs application in clinical practices is *PCA3* (Prostate cancer gene 3), an FDA-approved biomarker for the prostate cancer. According to this test, the *PSA* and *PCA3* RNA molecules are measured, and then the PCA3 Score (a ratio of *PCA3* RNA to *PSA* RNA) is calculated (http://www.fda.gov). Circulating lncRNAs have been also suggested as potential biomarkers for cancer and some other diseases. Exomal lncRNAs have been suggested as potential biomarkers to diagnose the malignant state of patients with prostate cancer (Işin et al.).

The rapidly growing list of ncRNAs holds promises that miRNAs and lncRNAs will become ever more important in illness management in future. The elucidation of the mechanisms by which lncRNAs act will help us to design suitable biomarkers or therapeutic agents for some diseases, especially cancer. The deciphering of the genetic networks and pathways regulated by the abnormally expressing lncRNAs, the characterization of miRNA-mRNA-lncRNA interaction, and the genetic/epigenetic mechanisms regulating the lncRNA expression provide more insights into the application of lncRNA research to human health. Considering the different molecular functions of ncRNAs, these RNAs can be the subject of a large diversity of further studies on complex diseases. We trust that the publications and the novelties on this subject will be exponentially extended in future.

## Author contributions

All authors listed, have made substantial, direct, and intellectual contribution to the work, and approved it for publication.

### Conflict of interest statement

The authors declare that the research was conducted in the absence of any commercial or financial relationships that could be construed as a potential conflict of interest.
